# Handedness Matters for Motor Control But Not for Prediction

**DOI:** 10.1523/ENEURO.0136-19.2019

**Published:** 2019-06-06

**Authors:** James Mathew, Fabrice R. Sarlegna, Pierre-Michel Bernier, Frederic R. Danion

**Affiliations:** 1 Aix Marseille Univ, CNRS, INT, Institut Neurosci Timone, 13005 Marseille, France; 2 Aix Marseille Univ, CNRS, ISM, 13009 Marseille, France; 3 Département de Kinanthropologie, Université de Sherbrooke, Sherbrooke J1k 2R1, Québec, Canada

**Keywords:** eye–hand coordination, hand dominance, humans, internal model, visuomotor tracking

## Abstract

Skilled motor behavior relies on the ability to control the body and to predict the sensory consequences of this control. Although there is ample evidence that manual dexterity depends on handedness, it remains unclear whether control and prediction are similarly impacted. To address this issue, right-handed human participants performed two tasks with either the right or the left hand. In the first task, participants had to move a cursor with their hand so as to track a target that followed a quasi-random trajectory. This hand-tracking task allowed testing the ability to control the hand along an imposed trajectory. In the second task, participants had to track with their eyes a target that was self-moved through voluntary hand motion. This eye-tracking task allowed testing the ability to predict the visual consequences of hand movements. As expected, results showed that hand tracking was more accurate with the right hand than with the left hand. In contrast, eye tracking was similar in terms of spatial and temporal gaze attributes whether the target was moved by the right or the left hand. Although these results extend previous evidence for different levels of control by the two hands, they show that the ability to predict the visual consequences of self-generated actions does not depend on handedness. We propose that the greater dexterity exhibited by the dominant hand in many motor tasks stems from advantages in control, not in prediction. Finally, these findings support the notion that prediction and control are distinct processes.

## Visual Abstract

**Figure:**
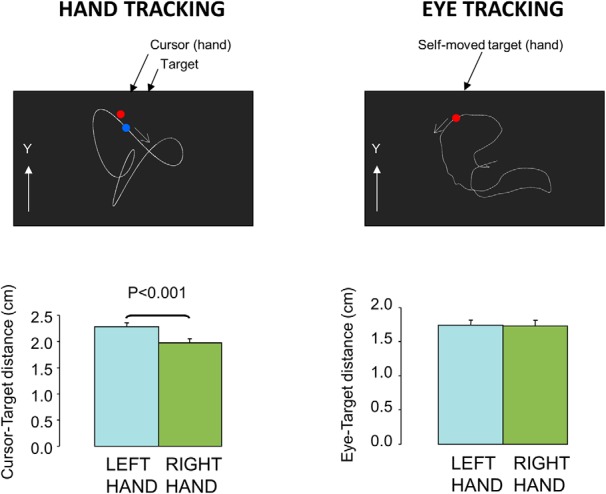

## Significance Statement

Humans often exhibit greater manual dexterity with the dominant hand. Here we assessed whether handedness similarly impacts control and prediction, two key processes for skilled motor behavior. Using two eye–hand coordination tasks that differently rely on control and prediction, we show that, although handedness impacts the accuracy of hand movement control, it has virtually no influence on the ability to predict the visual consequences of hand movements. We propose that the superior performance of the dominant hand stems from advantages in control, not in prediction. In addition, these findings provide further evidence that prediction and control are distinct neural processes.

## Introduction

Skilled motor behavior relies on the brain learning both to control the body and predict the consequences of this control (Flanagan et al., 2003). Control turns desired consequences into motor commands, whereas prediction turns motor commands into expected sensory consequences (Kawato, 1999; Wolpert et al., 2011; Shadmehr, 2017). Although there is ample evidence that manual dexterity depends on handedness, it remains unclear whether the superiority of the dominant hand stems from more efficient control and/or predictive mechanisms. Here, two eye–hand coordination tasks, known to rely differently on control and prediction were used to determine if these two processes are similarly influenced by handedness.

Motor control is generally more efficient for the dominant hand than the non-dominant hand. This idea is supported by numerous reports comparing the time to complete tests of manual dexterity (Bryden and Roy, 2005; Noguchi et al., 2006; Wang et al., 2011), as well as reports comparing the accuracy and variability of reaching movements (Carson et al., 1993; Elliott et al., 1993; Roy et al., 1994; Carey and Liddle, 2013; Schaffer and Sainburg, 2017). As for the effect of handedness on predictions, however, this issue has been less explored. Nonetheless, indirect evidence hints at the possibility that prediction could also be superior for the dominant hand. For instance it has been suggested that dominant hand movements rely on a better prediction of intersegmental dynamics (Sainburg and Kalakanis, 2000; Pigeon et al., 2013; Sainburg, 2014). Similarly, motor imagery, known to engage predictive mechanisms (Kilteni et al., 2018), has been shown to be more accurate for the dominant hand (Gandrey et al., 2013).

To assess whether the effect of handedness differs for control and prediction of hand movements, we tested right-handed participants on two types of eye–hand coordination tasks, each task being completed either by the right or the left hand. The first task was a hand-tracking task designed to assess the ability of participants to control their hand movement along an imposed trajectory (Carey et al., 1994; Foulkes and Miall, 2000; Sarlegna et al., 2010; Aoki et al., 2016; Moulton et al., 2017). During this task, participants had to control a cursor by means of a joystick so as to track a visual target that followed an unpredictable trajectory (Ogawa and Imamizu, 2013; Mathew et al., 2018). The second task was an eye-tracking task designed to test the ability of participants to predict the visual consequences of their hand movements. This time, participants were required to track with the eyes a target that was moved by their hand (Vercher et al., 1996; Landelle et al., 2016; Danion et al., 2017; Mathew et al., 2017). Such eye tracking of a self-moved target is known to rely on predictive mechanisms, supposedly based on the hand efference copy (Steinbach and Held, 1968; Scarchilli et al., 1999) as evidenced by the reduced temporal lag between eye and target position compared with eye tracking a target that is moved by an external agent (Steinbach and Held, 1968; Gauthier and Hofferer, 1976; Domann et al., 1989; Vercher et al., 1996).

In line with a large body of literature on arm reaching movements (Carson et al., 1993; Elliott et al., 1993; Roy et al., 1994; Carey and Liddle, 2013), previous studies have shown that the dominant (right) hand is more accurate for tracking a continuously moving target (Simon et al., 1952; Aoki et al., 2016; but see Carey et al., 1994; Moulton et al., 2017 ). We thus hypothesized that hand tracking, which reflects control, would be more accurate with the dominant hand. However, to our knowledge the possible influence of handedness on eye tracking a self-moved target has never been explored. In previous studies investigating this task, only the right dominant hand was used (Vercher et al., 1993, 1996; Scarchilli and Vercher, 1999; Chen et al., 2016a; Landelle et al., 2016; Danion et al., 2017; Mathew et al., 2017, 2018) or no (or incomplete) information was provided regarding participants’ handedness or the hand used in the task (Steinbach and Held, 1968; Steinbach, 1969; Gauthier and Hofferer, 1976; Gauthier et al., 1988). To date, we are only aware of a single study in which dominant and non-dominant hands were used (Chen et al., 2016b), but the putative impact of handedness was not reported.

## Methods

### Participants

Twenty-eight healthy right-handed volunteers (mean ± SD age, 26.6 ± 5.4 years; 13 females) were recruited. Handedness of participants was verified using the Oldfield Handedness Inventory (Oldfield, 1971) with a mean laterality quotient of 87.5 ± 12.9%. The experimental paradigm (2016-02-03-007) was approved by the local ethics committee of Aix-Marseille University and complied with the Declaration of Helsinki. All participants gave written consent before participation.

### Apparatus

**Figure 1.**
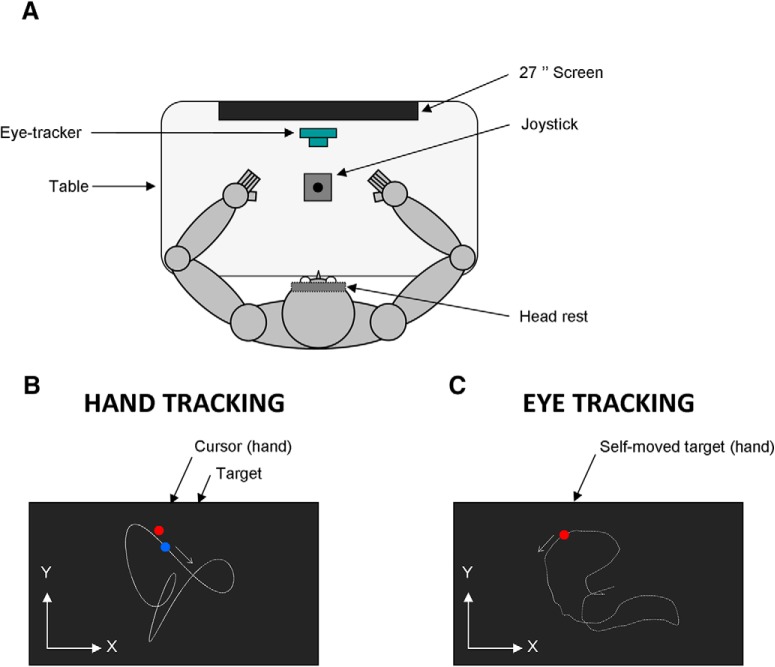
Schematic drawing of the experimental setup. ***A***, Top view of the participant sitting in the experimental setup. ***B***, Schematic view of the screen during the hand tracking condition. ***C***, Schematic view of the screen during the eye tracking condition (see Materials and Methods). The target trajectory (white dotted trace) and *x*–*y* reference system is displayed for illustration purposes but was not visible to the participant.

Figure 1 shows the experimental setup. Participants were comfortably seated in a dark room facing a screen (Benq, 1920 × 1080 pixels, 27 inches, 144 Hz) positioned in the frontal plane 57 cm away from their eyes. Note that 1° of visual angle is approximately equivalent to a distance of 1 cm on the screen at an eye-to-screen distance of 57 cm. Participants’ head movements were restrained by a chin rest and a padded forehead rest so that the eyes in primary position were directed toward the center of the screen. Both right and left forearms were resting on the table. To prevent vision of their hands, a piece of cardboard was positioned under the participants’ chin. Participants were required to hold with the hand a joystick (812 series, Megatron; with 25° of inclination along the *x*- and *y*-axes with no force bringing it back to the central position). The analog output of the joystick was sent to a data acquisition system (Keithley ADwin Real Time, Tektronix) and sampled at 1000 Hz.

Eye movements were recorded using an infrared video-based eye tracker (EyeLink 1000 Desktop, SR Research). Horizontal and vertical positions of the right eye were recorded at a sampling rate of 1000 Hz. The output from the eye tracker was calibrated before every block of trials by recording the raw eye positions as participants fixated a grid composed of nine known locations. The mean values during 1000 ms fixation intervals at each location were then used off-line for converting raw eye data to horizontal and vertical eye position in degrees of visual angle.

### Procedure

Participants performed one of two tracking tasks. In the hand-tracking task, participants had to move the joystick with their hand, so as to bring the cursor (red disk, 0.5 cm diameter) as close as possible to the target (blue disk, 0.5 cm in diameter) moving along a predefined trajectory. This task was used to probe the ability to control hand movements along an imposed trajectory (Tong and Flanagan, 2003; Ogawa and Imamizu, 2013; Mathew et al., 2018). The motion of the target resulted from the combination of sinusoids: two along the frontal axis (one fundamental and a second or third harmonic), and two along the sagittal axis (same procedure). The following equations determined the target’s motion:

$$x_{t}=A_{1x}\cos\omega t+A_{2x}\cos\left(h_{x}\omega t-\varphi_{x}\right)$$

$$y_{t}=A_{1y}\sin\omega t+A_{2y}\sin\left(h_{y}\omega t-\varphi_{y}\right).$$

**Figure 2.**
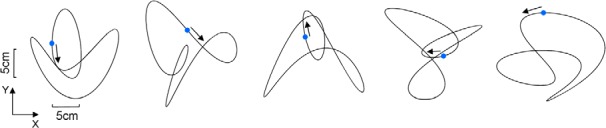
Target trajectories used during the hand-tracking task. The blue dot shows the initial position of the target, and the arrow shows its initial direction (see Materials and Methods).

This technique was used to generate pseudorandom 2D patterns while preserving smooth changes in velocity and direction (Mrotek and Soechting, 2007; Soechting et al., 2010). A total of five patterns with identical lengths were used throughout the experiment (Table 1; Fig. 2). All trajectories had a period of 5 s (fundamental = 0.2 Hz). During this task, participants did not receive any explicit constraints regarding their gaze, meaning they were free to look at the target, the cursor, or both (Danion and Flanagan, 2018).

**Table 1.** Target trajectory parameters in the hand-tracking task

Trajectory	*A1x*, cm	*A2x*, cm	Harmonic *x*	Phase *x*, °	*A1y*, cm	*A2y*, cm	Harmonic *y*	Phase *y*, °
1	5	5	2	45	5	5	3	−135
2	4	5	2	−60	3	5	3	−135
3	4	5.1	3	−60	4	5.2	2	−135
4	5	5	3	90	3.4	5	2	45
5	5.1	5.2	2	−90	4	5	3	22.5

In the eye-tracking task, participants were instructed to voluntarily move the joystick held in one hand so as to move a cursor (red disk, 0.5 cm in diameter) on the screen while concurrently keeping their eyes as close as possible to the cursor, which was thus a self-moved target. This task was used to probe the ability to predict the visual consequences of one’s hand movement (Vercher et al., 1995; Chen et al., 2016a; Landelle et al., 2016; Danion et al., 2017). Constraints were given with regard to the target (and thus hand) movement. First, participants were asked to generate random movements so as to make target motion as unpredictable as possible (Steinbach and Held, 1968; Landelle et al., 2016; Mathew et al., 2017). To facilitate the production of random movements, a template was provided on the screen during demonstration trials. Second, to maintain consistency across participants and trials, we ensured that, for each trial, mean tangential target velocity was close to 16 cm/s (thereby preserving task difficulty). This was done by computing mean target velocity online and by providing participants with verbal feedback during the experimental trials such as “please move faster” or “please slow down” when necessary. This procedure ensured minimal changes in mean target velocity across participants, trials, and hands. Participants were encouraged to cover the whole extent of the screen.

For both eye and hand-tracking tasks, we employed a fixed mapping between the joystick motion and the cursor motion with 25° of joystick inclination resulting in 15 cm on the screen. This mapping was such that a rightward/leftward hand motion corresponded to a rightward/leftward cursor motion, and a forward/backward hand motion corresponded to an upward/downward cursor motion. The duration of a trial was 10 s for both the eye- and hand-tracking tasks.

Participants were split into two groups that either performed the eye- or the hand-tracking task. One group of participants (*N* = 14, 8 males, mean age = 25.4 ± 4.0) performed the hand-tracking task, which consisted of one block of 10 trials with one hand followed by another 10 trial block with the other hand. Half of the participants started with the right hand. The second group of participants (*N* = 14, 7 males, mean age = 27.9 ± 6.4) followed the same type of protocol but with the eye-tracking task, i.e., each participant performed a block of 10 trials with each hand. Similarly, half of the participants started with the right hand. Before the beginning of the experiment, each participant performed a few practice trials (2 or 3) to familiarize with the task. Separate groups of participants were tested for hand and eye tracking because learning can transfer across these two tasks (Mathew et al., 2018).

To ensure that the eye-tracking task relied on predictive mechanisms, some participants of the second group (*N* = 10) completed 10 more trials in which they were asked to track with their eyes the target trajectories they had previously generated with their hand. During those trials, for each participant, we played back the last five target trajectories that he or she had generated with the right and left hand (Angel and Garland, 1972; Landelle et al., 2016; Mathew et al., 2017). Not only did this procedure allow for within-participant comparisons, it also minimized possible effects due to changes in target kinematics. The original order of trial presentation was maintained for each participant. We reasoned that if predictive mechanisms linking hand and eye actions are engaged when eye tracking the self-moved target, eye tracking of a self-moved target should be more accurate than eye tracking of a target, which follows the same trajectory but is moved by an external agent (Vercher et al., 1995; Landelle et al., 2016; Mathew et al., 2017).

### Data analysis

To assess hand-tracking performance, the following dependent variables were computed for each trial. First, we measured the mean Euclidian distance between the cursor (moved by hand) and the externally moved target (Gouirand et al., 2019). Second, we evaluated the time lag between the cursor and the target by means of cross-correlations (Danion et al., 2017). This procedure was conducted separately for the vertical and the horizontal axes, and the resulting lags were then averaged. To assess eye-tracking performance, the following dependent variables were computed from each trial. First, we measured the mean Euclidian distance between the eye and the self-moved target (Mathew et al., 2018). Second, we evaluated the time lag between gaze and target using the method described above. For all analyses, the first second of each trial was discarded.

To gain more insight about gaze behavior in both tasks, a sequence of analyses was performed to separate periods of smooth pursuit, saccades and blinks (Landelle et al., 2016; Danion et al., 2017; Mathew et al., 2017). The identification of the blinks was performed based on the pupil diameter (that was also recorded). This procedure led to the removal of 0.3% of eye recordings. Eye position time series in *x*- and *y*-axes were then separately low-pass filtered with a Butterworth (4th order) using a cutoff frequency of 25 Hz. The resultant eye position signals were differentiated to obtain the velocity traces. Tangential eye velocity was calculated from velocity traces in *x*- and *y*-axes. The eye velocity signals were low-pass filtered (Butterworth, 4th order, cutoff frequency: 25 Hz) to remove the noise from the numerical differentiation. The resultant eye velocity signals were then differentiated to provide the acceleration traces that were also low-pass filtered (Butterworth, 4th order, cutoff frequency: 25 Hz). Saccades were identified based on the acceleration and deceleration peaks (>1500 cm/s^2^). Further visual inspection allowed to identify smaller saccades (<1 cm) that could not be identified automatically by our program. Based on these computations, we evaluated for each trial the mean rate and amplitude of catch-up saccade, as well as the gain of smooth pursuit in both tasks (Mathew et al., 2017; Danion and Flanagan, 2018).

To provide more information about the dynamics of the tracking error in both tasks, power spectral analyses of the hand-target and eye-target distance were performed in the 0–5 Hz frequency range. To assess whether the complexity of hand/target motion was similar for the right and left hand during the eye-tracking task, approximate entropy (ApEn) was used as an index to characterize the unpredictability of a signal (Pincus, 1991); the larger the approximate entropy the more unpredictable the signal is. To compute approximate entropy we used the following MATLAB function: https://fr.mathworks.com/matlabcentral/fileexchange/32427-fast-approximate-entropy [with the following settings: embedded dimension = 2, tolerance = 0.2 × STD(target trajectory)]. Approximate entropy was measured separately on the *x*- and *y*-axes.

### Statistics

Paired *t* tests and repeated-measures ANOVAs were used to assess the effects of HAND (i.e., Right/Left), FREQUENCY, and AGENCY (Self/External). Newman–Keuls *post hoc* tests were used whenever needed. Kolmogorov–Smirnov tests showed that none of the dependent variables significantly deviated from a normal distribution. A 0.05 significance threshold was used for all analyses.

## Results

### Typical trials

**Figure 3.**
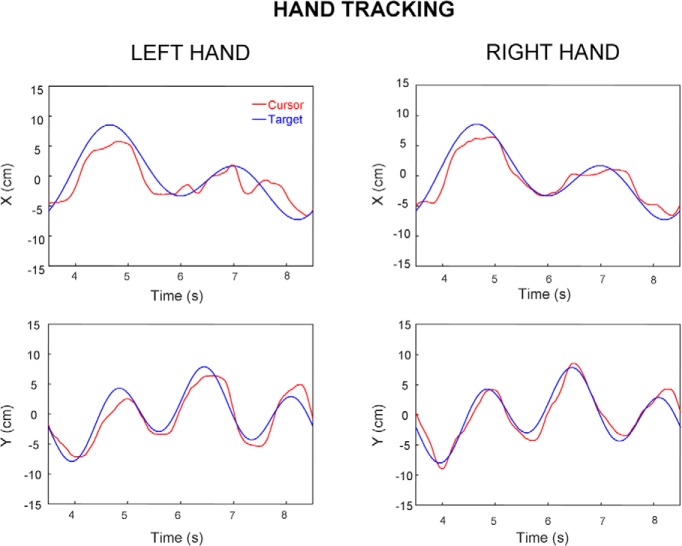
Typical portions of hand tracking trials performed by the same participant with the same target trajectory. Left and right columns, respectively, display the performance of left and right hands. Top and bottom rows, respectively, display the horizontal and vertical components of hand (cursor, red) and target (blue) movement. The cursor is generally closer to the target when being moved by the right hand compared with the left hand.

Figure 3 plots two representative portions of trials performed by one right-handed participant who tracked the visual target either with the right or the left hand. As can be seen, this figure suggests that hand tracking was more accurate when using the right (dominant) hand.

**Figure 4.**
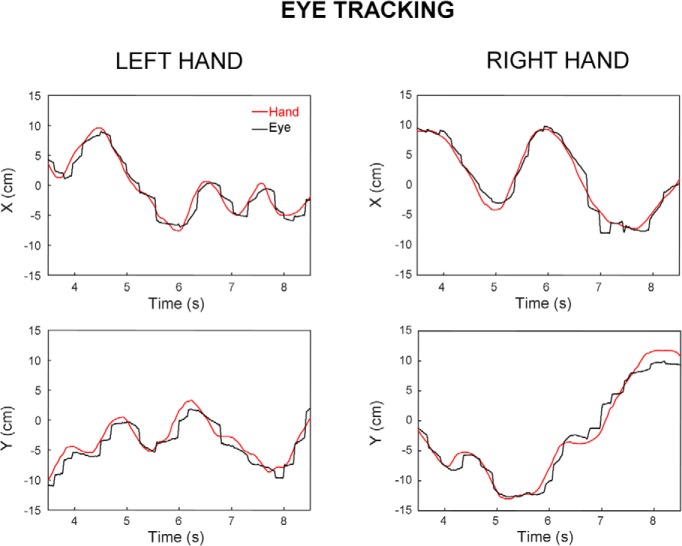
Typical portions of eye tracking trials performed by the same participant. Left and right columns, respectively, display eye-tracking performance when moving the target either with the left or right hand. Top and bottom rows, respectively, display the horizontal and vertical components of hand (self-moved target; red) and eye (black) movement.

Figure 4 shows two representative portions of trials performed by another right-handed participant that had to track with the eyes a target moved either by the right (right column) or left hand (left column). In this case, visual inspection does not suggest any evident difference in eye tracking accuracy across hands. In the next sections, we analyze in more details the possible effect of handedness on eye and hand tracking across all participants.

### Hand tracking is more accurate with the dominant hand

**Figure 5.**
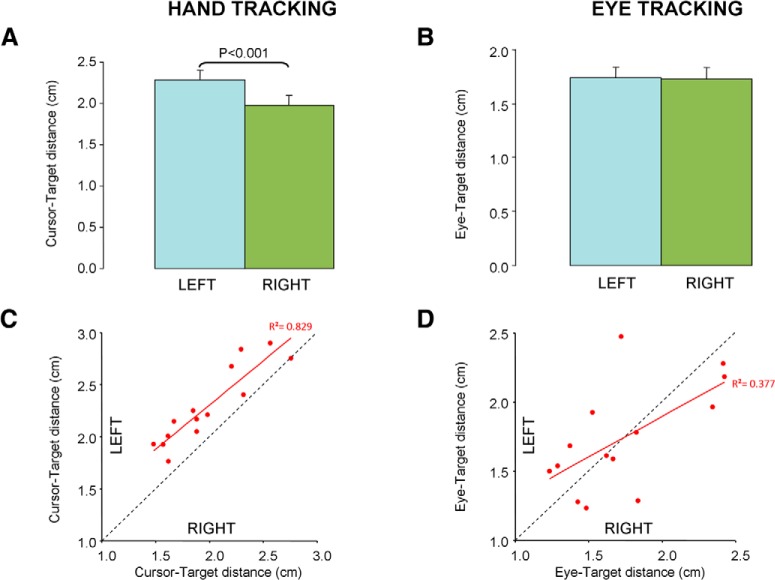
Effect of handedness on tracking accuracy. ***A***, Mean group hand tracking error when tracking the target with the right or the left hand. Error bars represent the standard error of the mean. ***B***, Same as ***A*** for eye-tracking error. ***C***, Correlation between right and left hand-tracking performance. Each red dot represents one participant. The red line indicates the linear regression, and the dotted black line indicates equality between right and left hand. ***D***, Same as ***C*** for eye tracking when moving the target with either the right or the left hand.

Mean data showed that right-handed participants tracked the target more accurately with the right than the left hand (Fig. 5*A*). On average, the cursor-target distance was 16% larger when using the left hand (2.29 ± 0.39 vs 1.98 ± 0.37 cm; *t*_(13)_ = 6.96; *p* < 0.001). Figure 5*C* shows that this difference was quite systematic across participants, and also that the accuracies of the right and left hand were correlated across participants (*R* = 0.91; *p* < 0.001). Regarding the temporal relationship between cursor and target, the lag did not significantly differ between the right and left hands (70 vs 77 ms; *t*_(13)_=1.41; *p* = 0.18), and those lags were correlated across participants (*R* = 0.83; *p* < 0.001).

**Figure 6.**
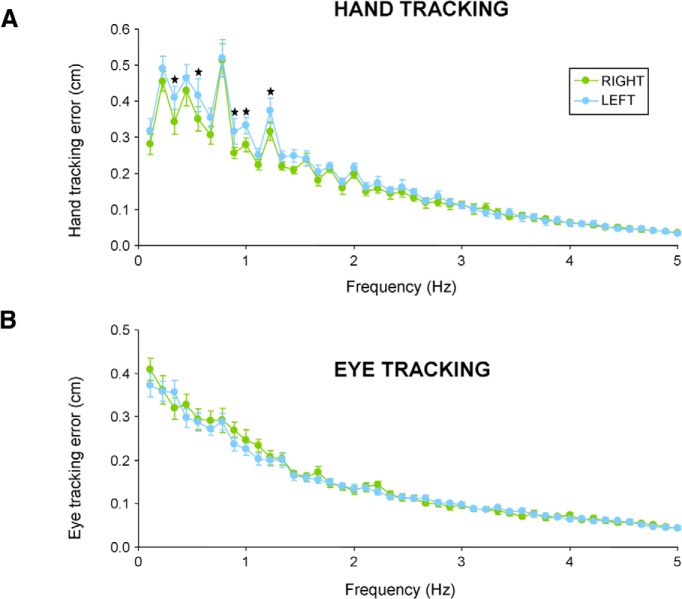
Effect of handedness on the power spectrum of tracking error in each task. ***A***, Power spectrum of cursor-target distance during hand tracking. ***B***, Power spectrum of eye-target distance during eye tracking. Error bars represent the standard error of the mean. Black stars indicate frequency bin in which a significant difference across hands was observed (*p* < 0.05).

Figure 6*A* presents the corresponding power spectrum of hand tracking error as a function of hand. A two-way ANOVA with FREQ (45 levels: 0.11–5 Hz with 0.11 Hz step) and HAND showed a main effect of HAND (*F*_(1,13)_=10.2; *p* < 0.01), as well as an effect of FREQ (*F*_(44,572)_=74.76; *p* < 0.001) and an interaction between the two (*F*_(44,572)_=1.7; *p* < 0.01). *Post hoc* analysis of the interaction showed that bins in which hand-tracking errors were larger with the left hand were in the 0.3–1.2 Hz frequency range.

Further analyses were conducted to examine whether those differences in hand tracking accuracy were associated with different gaze behaviors. *T* tests showed no significant differences between gaze behaviors when tracking the target with the right or left hand, neither in terms of eye-target distance (1.50 vs 1.54 cm; *t*_(13)_=0.74; *p* = 0.47), nor in terms of saccade rate (2.72 vs 2.68 sac/s; *t*_(13)_=0.49; *p* = 0.63), saccade amplitude (2.0 vs 2.0 cm; *t*_(13)_=0.16; *p* = 0.87) or even smooth-pursuit gain (0.82 vs 0.82; *t*_(13)_=0.68; *p* = 0.51). We conclude that the greater accuracy of the right hand for manual tracking does not stem from a better monitoring of target motion by the eyes.

### Handedness does not influence eye tracking of a self-moved target

In contrast to hand tracking, participants exhibited similar levels of performance in eye tracking when moving the target with the right or left hand (Fig. 5*B*). Indeed we found no significant difference in tracking accuracy across hands (*t*_(13)_=0.11; *p* = 0.92) with mean group eye-target distance being respectively 1.73 ± 0.40 and 1.74 ± 0.39 cm when using the right or left hand. The accuracy of eye tracking when using the right and left hand was correlated across participants (*R* = 0.61; *p* = 0.01; Fig. 5*D*). Regarding the temporal relationship between eye and target, we found that the eye followed the target by ∼40 ms but the lags for the right and left hands did not significantly differ (41 vs 45 ms; *t*_(13)_=1.30; *p* = 0.22), and were correlated with each other (*R* = 0.57; *p* < 0.05).

Similar gaze strategies appeared to be used with both hands. Indeed *t* tests showed no significant effects of HAND for smooth-pursuit gain (0.62 vs 0.63; *t*_(13)_=1.25; *p* = 0.23), saccade rate (3.03 vs 3.15 sac/s; *t*_(13)_=1.41; *p* = 0.18), and saccade amplitude (2.0 vs 2.1 cm; *t*_(13)_=1.08; *p* = 0.30). For all these dependent variables, the correlation between hands was significant (each *R* > 0.64, each *p* < 0.01). Analysis of target motion randomness by means of approximate entropy along either the *x*- or *y*-axis showed no significant effect of HAND (each *t*_(13)_<1.64, *p* > 0.12). Further analyses of mean target tangential velocity also failed to show a significant difference across hands (15.9 vs 15.9 cm/s; *t*_(13)_=0.05; *p* = 0.96).

Regarding FFT analyses of eye tracking error, Figure 6*B* presents the corresponding power spectrum associated with each hand. A two-way ANOVA showed a main effect of FREQ (*F*_(44,572)_=125.45; *p* < 0.001) but no significant main effect of HAND (*F*_(1,13)_=0.36; *p* = 0.55) and no significant interaction between FREQ and HAND (*F*_(44,572)_=1.03; *p* = 0.41). These results further support the view that eye tracking had similar dynamics when moving the target with the right or the left hand. Overall eye tracking was rather insensitive to which hand was used to move the target.

The lack of significant differences across hands in the eye-tracking task should not automatically lead to the conclusion that handedness does not influence eye tracking of a self-moved target. To quantify how true the null hypothesis may be, we used Bayesian statistics with the JASP free software (https://jasp-stats.org). Repeating the previous *t* tests with the Bayesian approach led to BF_10_ scores that ranged between 0.27 and 0.62, providing from substantial to anecdotal evidence in favor of the null hypothesis (Lee and Wagenmakers, 2014). None of these Bayesian *t* tests provided evidence for the alternative hypothesis.

### *Additional evidence that prediction underlies eye tracking of a self-moved target: self-moved* versus *externally-moved target*

**Figure 7.**
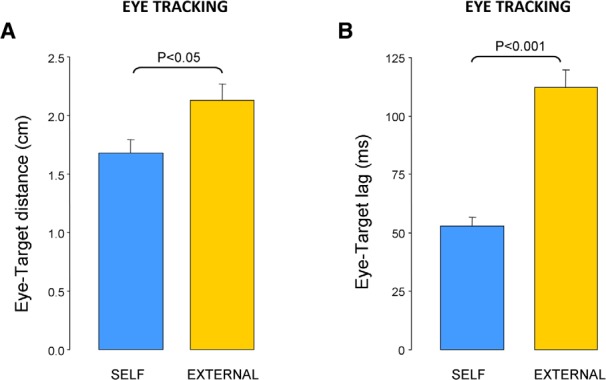
Comparison between eye tracking a self-moved target and an externally moved target. ***A***, Effect of agency on eye-target distance. ***B***, Effect of agency on eye-target lag. Error bars represent SEM.

For comparison purposes, 10 participants of the eye-tracking group were also asked to track with their eyes target trajectories that each of them had previously generated during the self-moved condition. Figure 7 shows that eye-tracking performance was less accurate in those playback trials with an externally-moved target than those in which they moved the target themselves. This view was confirmed by a two-way ANOVA (AGENCY×HAND) showing a main effect of AGENCY (*F*_(1,9)_=6.59; *p* < 0.05) on eye-target distance, which was 27% larger during trials with an externally-moved target than during self-moved trials (2.13 vs 1.68 cm; Fig. 7*A*). There was no significant effect of HAND (*F*_(1,9)_=0.10; *p* = 0.75), or interaction between HAND and AGENCY (*F*_(1,9)_=0.16; *p* = 0.69). Similar results were obtained when analyzing the eye-target lag (Fig. 7*B*) as we found a main effect of AGENCY (*F*_(1,9)_=51.06; *p* < 0.001) showing a twofold increase in the eye-target lag in playback trials with an externally-moved target compared with self-moved trials (112 vs 53 ms, respectively). There was no significant effect of HAND (*F*_(1,9)_=1.82; *p* = 0.21) or interaction (*F*_(1,9)_=2.00; *p* = 0.19). These results are consistent with the idea of predictive mechanisms linking eye and hand actions when participants have to track a self-moved target.

## Discussion

Our main objective was to tease apart the possible effect of handedness on prediction and control of hand movements. To achieve this objective, we investigated interlimb differences when performing either a hand tracking or an eye-tracking task. Our main observation is that, in contrast to hand tracking that was clearly impacted by handedness, eye tracking was nearly identical irrespective of whether the target was moved by the right or the left hand. We now propose to discuss in more detail these findings and their implications for prediction and control of hand movements.

### Handedness matters for hand tracking

We found that when asked to move a cursor along an imposed trajectory, right-handed participants were more accurate when using their right (dominant) hand compared with the left (non-dominant) hand. Indeed, as shown by our analyses, the cursor-target distance was lower when participants used their right hand. Our FFT analyses further confirmed the superiority of the right hand with lower tracking error between 0.3 and 1.2 Hz, a frequency range that matches with rather slow (voluntary) visuomotor feedback loops. Overall these results are consistent with previous studies that explored the effect of hand dominance during hand tracking (Simon et al., 1952; Carey et al., 2003; Aoki et al., 2016), as well as other studies investigating reaching movements (Carson et al., 1993; Elliott et al., 1993; Roy et al., 1994; Carey and Liddle, 2013; Schaffer and Sainburg, 2017), and conventional tests of manual dexterity (Bryden and Roy, 2005; Noguchi et al., 2006).

Despite clear differences in hand tracking accuracy, there were strong correlations between the right and left hand behavior across participants, both in terms of cursor-target distance and cursor-target lag. Our observations echo another study showing that the consistency of hand reaching movements is correlated across hands (Haar et al., 2017b). Altogether, these observations suggest that the neural circuits driving right and left hand actions are coupled to some extent. This coupling across hands can stem from various factors including visual perception, motivation/arousal, and decisional/planning processes.

Because during hand tracking, gaze is related more closely to the target than the cursor (Danion and Flanagan, 2018), it was crucial to assess whether the asymmetry across hands could be explained by different gaze behaviors. Our analyses of gaze showed that neither the eye-target distance, nor the saccade rate, the saccade amplitude or the smooth-pursuit gain, were influenced by handedness. We conclude that the lower performance exhibited by the left hand does not stem from poorer processing of visual information about the target motion. Altogether, those results suggest that the ability to generate adequate hand motor commands to bring the cursor close to the moving target is better for the right hand. These findings thus extend the idea that there is a right hand advantage for trajectory control toward a stationary target (Sainburg and Kalakanis, 2000; Bagesteiro and Sainburg, 2002; Mutha et al., 2012) to the condition of a moving target.

### Handedness does not matter for eye tracking a self-moved target

We consistently found no significant difference in eye-tracking performance when moving the target with the right or the left hand. This view was supported by similar eye-target distance, eye-target lag, saccade rate, saccade amplitude, smooth pursuit gain, and spectral analyses of error. One possible confound could be that right hand motion was faster and/or more complex than left hand motion but we showed that mean target velocity, as well as randomness of target motion were similar for both hands, the latter observation being consistent with a report comparing the randomness of right and left finger movements (Newell et al., 2000). Finally, because one could argue that predictive mechanisms were not at play in our eye-tracking task, we performed additional trials demonstrating that eye-tracking performance was substantially improved when the target was self-moved compared with when it was externally moved, which fits with many other studies (Steinbach and Held, 1968; Vercher et al., 1995; Chen et al., 2016b; Landelle et al., 2016). Overall, our study suggests that the ability to predict visual consequences arising from voluntary hand actions does not depend on handedness. At first sight this conclusion may seem inconsistent with the idea of Sainburg et al. (1995) that the dominant hand has an advantage for predicting intersegmental torques (Yadav and Sainburg, 2014), but in our opinion this ability could also reflect a better inverse model of arm dynamics.

One may wonder to what extent increasing the difficulty of eye tracking a self-moved target could have been helpful to further tease apart the predictive mechanisms engaged for each hand. Pilot data collected when first exploring this task with the right hand (Landelle et al., 2016) showed that faster hand/target motion led to a drop in eye-tracking performance, making the involvement of predictive mechanisms less obvious (i.e., the difference between self-moved and externally-moved target conditions faded). Whether this drop in predictive performance induced by increasing task difficulty would be similar for both hands remains to be explored.

### Implications for control and prediction of the right and left hands: toward a possible scheme

**Figure 8.**
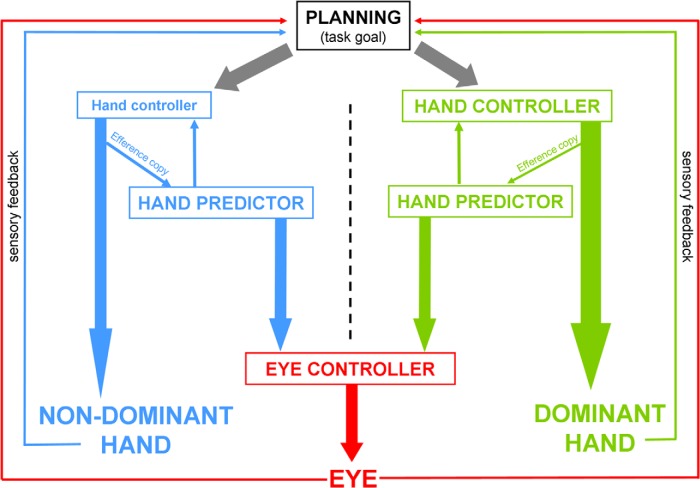
Possible scheme accounting for separate effects of handedness on hand tracking and eye tracking. High-level planning of cursor/target motion is effector independent, which may partly explain the correlated hand performances. Each hand is associated with a separate controller and predictor though. During eye tracking a self-moved target, the eye controller is fed by the predictor of the moving hand. Both predictors have a similar accuracy, resulting in similar performance when tracking with the eyes a target moved by the dominant (right) or non-dominant (left) hand. However, the controller of the dominant hand is more accurate, resulting in better performance when tracking a visual target with this hand.

The main goal of the study was to determine whether control and prediction are similarly influenced by handedness as we hoped to clarify whether the superiority of the dominant hand stems from more efficient control, prediction, or both. We found that right-handed participants were more accurate when using their right hand for hand tracking, an effect expected from the literature, but this right-hand advantage was not observed in the eye-tracking task. Moreover, we observed in each task that performance of the right and left hands were correlated such that if one participant had poor performance with one hand, he or she was likely to also exhibit poor performance with the other hand. In Figure 8 we propose a hypothetical scheme that could account for all these observations. Although this scheme is largely inspired from other accounts in which an inverse model (also called controller) and a forward model (also called predictor) contribute to hand movements (Kawato, 1999; Wolpert and Flanagan, 2001; Diedrichsen et al., 2010; Shadmehr et al., 2010; Scott, 2012), we propose to emphasize the possible difference between dominant and non-dominant hand actions.

A parsimonious explanation for better hand tracking with the dominant hand is that the controller (inverse model) in charge of this hand issues motor commands that allow reaching more adequately the desired (target) position. This possibility receives credit from several brain imaging studies showing a larger hand representation in the primary motor cortex of the dominant hemisphere (Triggs et al., 1994; Amunts et al., 1996; Volkmann et al., 1998; Hammond, 2002), a brain region often evoked as a possible site for an inverse model (Shadmehr and Krakauer, 2008; Scott, 2012). As for the correlation in performance across hands, this effect may arise from common visual processing of target motion (i.e., similar gaze behavior), motivational factors, as well as effector-independent planning linking ongoing cursor and target states to desired cursor motion (Medendorp et al., 2003), all taking place upstream from the computations of the motor commands issued by the inverse model. This correlation could also be supported by the fact that upper limb movements involve effector-independent representations in the contra and ipsilateral hemisphere (Haar et al., 2017a), as well as bilateral representations (Berlot et al., 2019).

As eye-tracking performance was similar across hands, a first option would be to consider that a single forward model is in charge of predicting the visual consequences of both hand movements. Such a shared forward model fed by higher-order signals, for instance hand direction in extrinsic coordinates at the planning stage (Crawford et al., 2004), would account for the lack of hand dominance effect. However, one problem with this scheme is that we observed only moderate correlation in eye-tracking performance across hands (especially compared with hand tracking, supposedly driven by separate controllers). As a result we favor the hypothesis that there are separate forward models in charge of predicting the visual consequences of each hand movement. In line with earlier suggestions (Steinbach and Held, 1968; Vercher et al., 1996; Scarchilli et al., 1999), we propose that these forward models are fed by the associated hand efference copy, a signal that could be issued upstream of the primary motor cortex (Voss et al., 2007; Mathew et al., 2017). In contrast with inverse models, our findings suggest that dominant and non-dominant forward models have a similar accuracy, meaning that their ability to predict the outcome of hand movements is not impacted by the correctness of the input signal. The fact that eye-tracking performance was correlated across hands suggests that these two forward models might not be fully independent of each other. Although brain regions such as the parietal cortex and the cerebellum have often been evoked for their contribution to sensory prediction (Blakemore and Sirigu, 2003; Pasalar et al., 2006; Miall et al., 2007; Mulliken et al., 2008; Shadmehr and Krakauer, 2008; Scott, 2012), lateralization and/or possible asymmetries in these structures remains poorly understood. Yet there is evidence that volume asymmetries in the cerebellum may depend on handedness (Ocklenburg et al., 2016; but see Snyder et al., 1995 ). Despite several evidences that the cerebellum is key for eye–hand coordination (Vercher and Gauthier, 1988; Miall et al., 2001), the possible structural asymmetry of the cerebellum did not seem to significantly influence eye-tracking performance.

The scheme presented in Figure 8 in which we hypothesize different controllers but similar predictors raises a question: why do participants exhibit worse hand-tracking performance with the left hand, if prediction is supposedly as accurate for right and left hand movements? It has been proposed that forward modeling provides internal feedback loops optimizing the accuracy of hand movements (Desmurget and Grafton, 2000), so why can’t the predictor of the left hand compensate for the putatively weaker controller of the left hand? We see several possible reasons. First, the eye-tracking task used in the current study suggests similar abilities to predict the visual consequences of right and left hand movements, but it remains unclear whether this finding extends to somatosensory consequences of right and left hand movements. This reasoning goes along with the proposition that the brain could predict separately the visual and the somatosensory consequences of actions (Miall et al., 1993) by using different neural populations (Liu et al., 2003). Moreover our eye-tracking task tested the ability of the eye to make use of predicted hand movements, but it did not explicitly test the internal feedback loops associated with the control of hand movements (Desmurget and Grafton, 2000). One possibility could be that in these two contexts, eye and hand rely differently on predictions made for visual and proprioceptive consequences of hand movement. In addition, one may hypothesize that in the current context in which the mapping between the cursor and the joystick is one-to-one (no perturbation), the coupling between the predictor and the controller is weaker than when adaptation is required (Honda et al., 2018).

### Final comments

Although it is usually difficult to tease apart the contribution of forward and inverse models (Lalazar and Vaadia, 2008; Mulliken et al., 2008), the current design allowed to unpack these contributions, and revealed an asymmetrical effect of handedness on prediction and control. What are the implications of this finding with respect to the greater dexterity exhibited by the dominant hand in a wide range of task? At this stage, our results suggest that the dominant hand advantage stems from better control, but not necessarily from better prediction. Although brain imaging studies have provided evidences for functional and structural asymmetries between the right and left hemispheres of the human brain (Hammond, 2002; Toga and Thompson, 2003), some of these being correlated with handedness (Kim et al., 1993; Elbert et al., 1995; Amunts et al., 1996), here we show that handedness does not impact the ability to predict visual consequences of hand actions. More generally these findings provide further evidence that prediction and control are distinct processes (Kawato, 1999; Flanagan et al., 2003; Shadmehr, 2017).

## Synthesis

Reviewing Editor: Gustavo Deco, Universitat Pompeu Fabra Decisions are customarily a result of the Reviewing Editor and the peer reviewers coming together and discussing their recommendations until a consensus is reached. When revisions are invited, a fact-based synthesis statement explaining their decision and outlining what is needed to prepare a revision will be listed below. The following reviewer(s) agreed to reveal their identity: Hiroshi Imamizu, Charalambos Papaxanthis.

Reviewer 1:

The authors examined whether handedness affects prediction of the visual consequence of hand movements. They made participants track with their eyes a target that was self-moved by their dominant or non-dominant hands. This task tests the ability to predict the visual consequence of hand movements from motor commands. Their results indicate that the ability to predict the visual consequences of self-generated actions does not depend on handedness. This suggests that greater dexterity exhibited by the dominant hand stems from advantages in control but not in prediction.

This is an interesting work that covers an overlooked issue in human motor control by clever combination of simple experiments. The manuscript is clearly and concisely written with respects to the introduction, method and result sections. However, there seems to be gaps between their results and conclusions that should be filled by the additional analysis or discussion.

1. The authors assume predictors separately for the left and right hands (e.g., Figure 8), and concluded no significant difference in performance between the two predictors. However, is there any possibility that there is only one predictor that predicts visual consequence from higher order signals, which are unspecific to the hands (such as a hand direction in the extrinsic coordinates at the planning stage), and that output signal from the single model is utilized by the eye controller?

2. Their conclusion is based on observation that there is a significant effect of handedness on accuracy of the hand tracking task but not the eye tracking task. However, there seems to be larger individual difference in the eye tracking task (Figure 5D) than the hand tracking task (Figure 5C). Is there any possibility that the difference affected the results?

Reviewer 2:

IIn the present study the authors examined whether on-line control and predictive processes are similarly impacted by manual dexterity. Right-handed participants performed two tasks either with the right or the left hand. In the first task, which tested the ability to control online the hand along an imposed trajectory, participants had to move a cursor with their and so as to track a target that followed a quasi-random trajectory. In the second task, which tested the ability to predict the visual consequences of hand movements, participants had to track with their eyes a target that was self-moved through voluntary hand motion. The main results showed that hand tracking was more accurate with the right hand than with the left hand. In contrast, eye tracking was similar in terms of spatial and temporal gaze attributes whether the target was moved by the right or the left hand. The authors propose that the greater dexterity exhibited by the dominant hand in many motor tasks stems from advantages in control, not in prediction.

Comments:

The study is interesting and original. The paper is well-written and the results are clearly presented.

One question is whether the hand-tracking and the eye-tracking tasks were of similar difficulty. Could the authors clarify this point? In the same line, do the authors think that differences between the two hands would appear (eye-tracking task) if hand trajectories were more complex and faster?

From a computational point of view, if predictive mechanisms do not differ between hands, and theoretically both hands can take advantage from similar internal correction processes, why control process is better for the right than the left hand? Is authors explanation about a better ‘inverse model’ for the right hand sufficient (lines 413-417)? This point merits further development in the discussion.

Please explicitly state that variables followed normal distribution.
